# Supplementary material for: Isolation, Identification and Chemical Modification of Bufadienolides from Bufo melanostictus Schneider and Their Cytotoxic Activities against Prostate Cancer Cells
Source: Molecules. 2024 Mar 31;29(7):1571. doi: 10.3390/molecules29071571 (PMC11013645; doi:10.3390/molecules29071571)
Supplement: Supplementary file 1 [file molecules-29-01571-s001.zip › molecules-2832695-supplementary.pdf]

Isolation, identification and chemical modification of bufadienolides from *Bufo melanostictus* Schneider and their cytotoxic activities against prostate cancer cells

Content

Figure S1. Spectra of undescribed compounds **1-2** isolated from toad venom 3
Figure S1.1. HR-ESI-MS spectrum of **1**....3
Figure S1.2. ^{1}H NMR spectrum of **1** (in CD_3OD, 400 MHz)....4
Figure S1.3. ^{13}C NMR spectrum of **1** (in CD_3OD, 100 MHz)....4
Figure S1.4. DEPT-135 spectrum of **1** (in CD_3OD, 100 MHz)....5
Figure S1.5. ^{1}H-^{1}H COSY spectrum of **1** (in CD_3OD) 5
Figure S1.6. HSQC spectrum of **1** (in CD_3OD)....6
Figure S1.7. HMBC spectrum of **1** (in CD_3OD) 6
Figure S1.8. NOESY spectrum of **1** (in CD_3OD)....7
Figure S1.9. HR-ESI-MS spectrum of **2**....7
Figure S1.10. ^{1}H NMR spectrum of **2** (in CD_3OD, 400 MHz) 8
Figure S1.11. ^{13}C NMR spectrum of **2** (in CD_3OD, 100 MHz)....8
Figure S1.12. DEPT-135 spectrum of **2** (in CD_3OD, 100 MHz) 9
Figure S1.13. ^{1}H-^{1}H COSY spectrum of **2** (in CD_3OD)....9
Figure S1.14. HSQC spectrum of **2** (in CD_3OD) 10
Figure S1.15. HMBC spectrum of **2** (in CD_3OD) 10
Figure S1.16. NOESY spectrum of **2** (in CD_3OD)....11
Figure S2. Spectra of four lactam (**14-17**) of bufadienolides by the conversion of lactone of bufadienolides....12
Figure S2.1. ^{1}H NMR spectrum of **14** (in CD_3OD, 600 MHz) 12
Figure S2.2. ^{13}C NMR spectrum of **14** (in CD_3OD, 150 MHz) 13
Figure S2.3. ^{1}H NMR spectrum of **15** (in CD_3OD, 600 MHz) 13
Figure S2.4. ^{13}C NMR spectrum of **15** (in CD_3OD, 150 MHz) 14
Figure S2.5. ^{1}H NMR spectrum of **16** (in CD_3OD, 600 MHz) 14
Figure S2.6. ^{13}C NMR spectrum of **16** (in CD_3OD, 150 MHz) 15
Figure S2.7. ^{1}H NMR spectrum of **17** (in CD_3OD, 600 MHz) 15
Figure S2.8. ^{13}C NMR spectrum of **17** (in CD_3OD, 150 MHz) 16
Table S1. ^{1}H (600 MHz) and ^{13}C (150 MHz) NMR spectroscopic data of **14** in CD_3OD 17
Table S2. ^{1}H (600 MHz) and ^{13}C (150 MHz) NMR spectroscopic data of **15** in CD_3OD 18
Table S3. ^{1}H (600 MHz) and ^{13}C (150 MHz) NMR spectroscopic data of **16** in CD_3OD 19
Table S4. ^{1}H (600 MHz) and ^{13}C (150 MHz) NMR spectroscopic data of **17** in CD_3OD 20

Figure S1. Spectra of undescribed compounds **1-2** isolated from toad venom

Figure S1.1. HR-ESI-MS spectrum of **1**

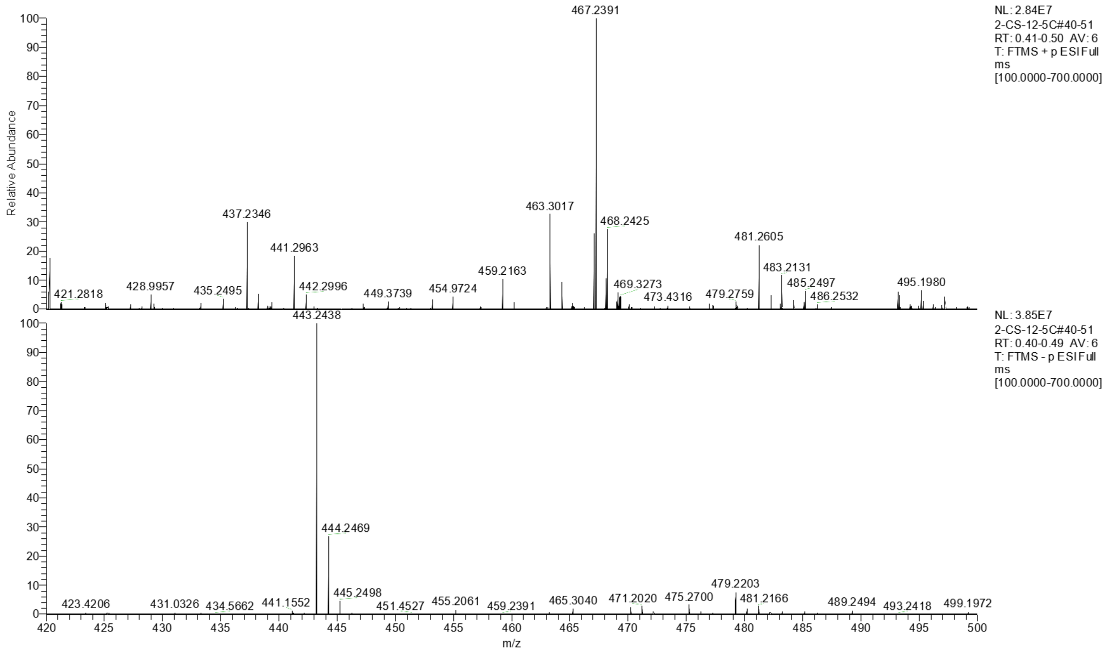

Figure S1.2. ^{1}H NMR spectrum of **1** (in CD_3OD, 400 MHz)

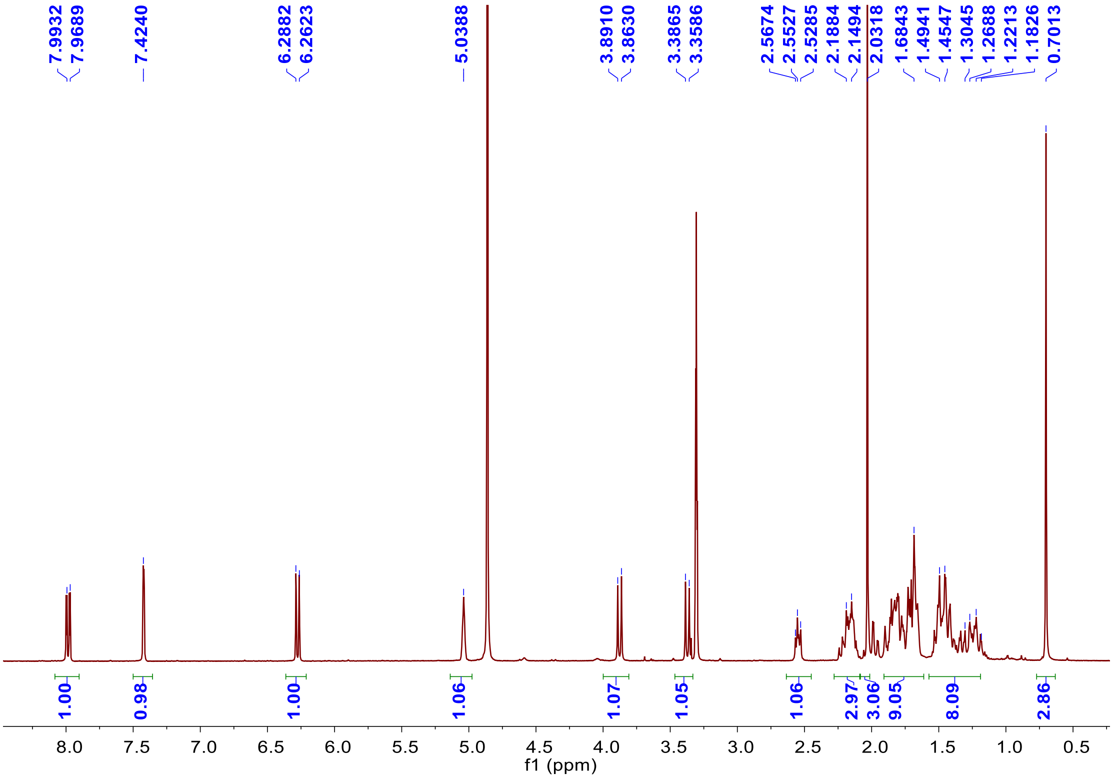

Figure S1.3. ^{13}C NMR spectrum of **1** (in CD_3OD, 100 MHz)

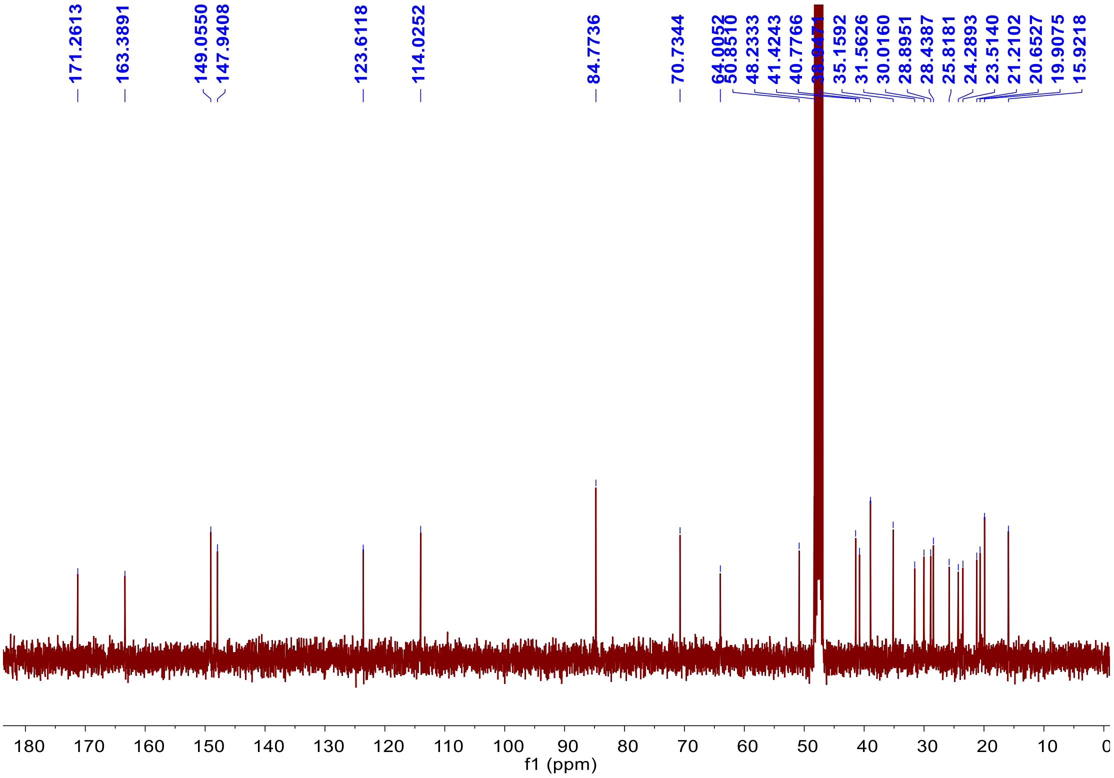

Figure S1.4. DEPT-135 spectrum of **1** (in CD_3OD, 100 MHz)

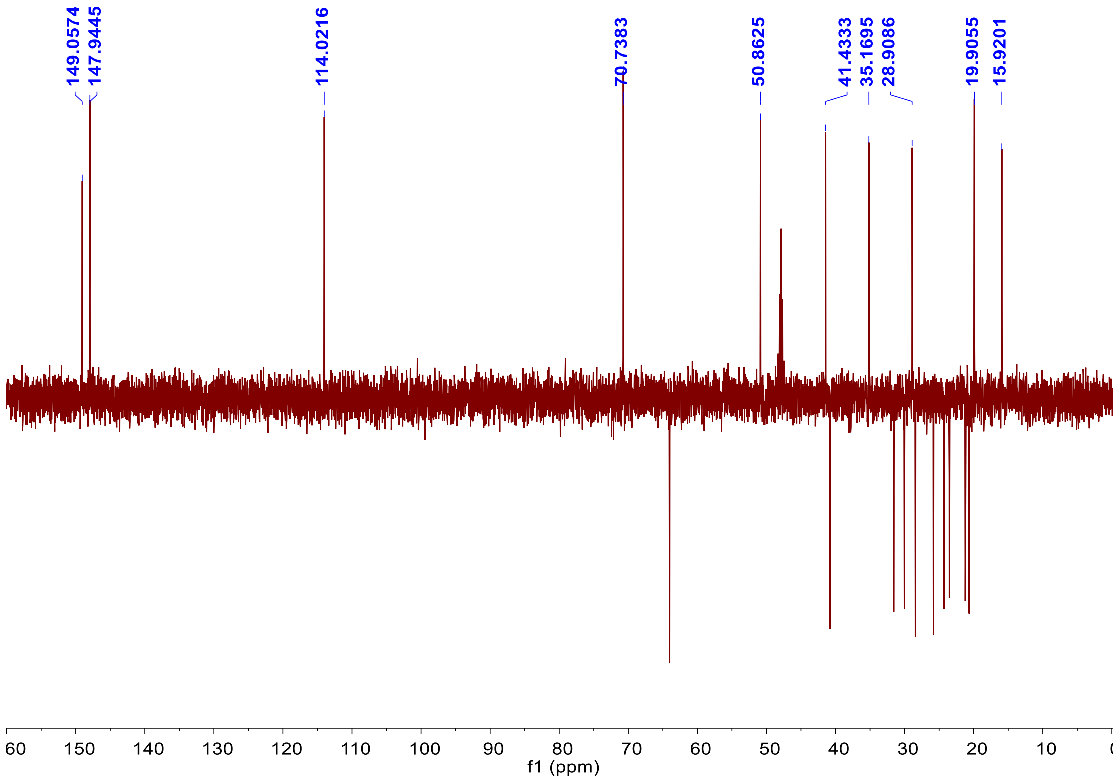

Figure S1.5. ^1H-^1H COSY spectrum of **1** (in CD_3OD)

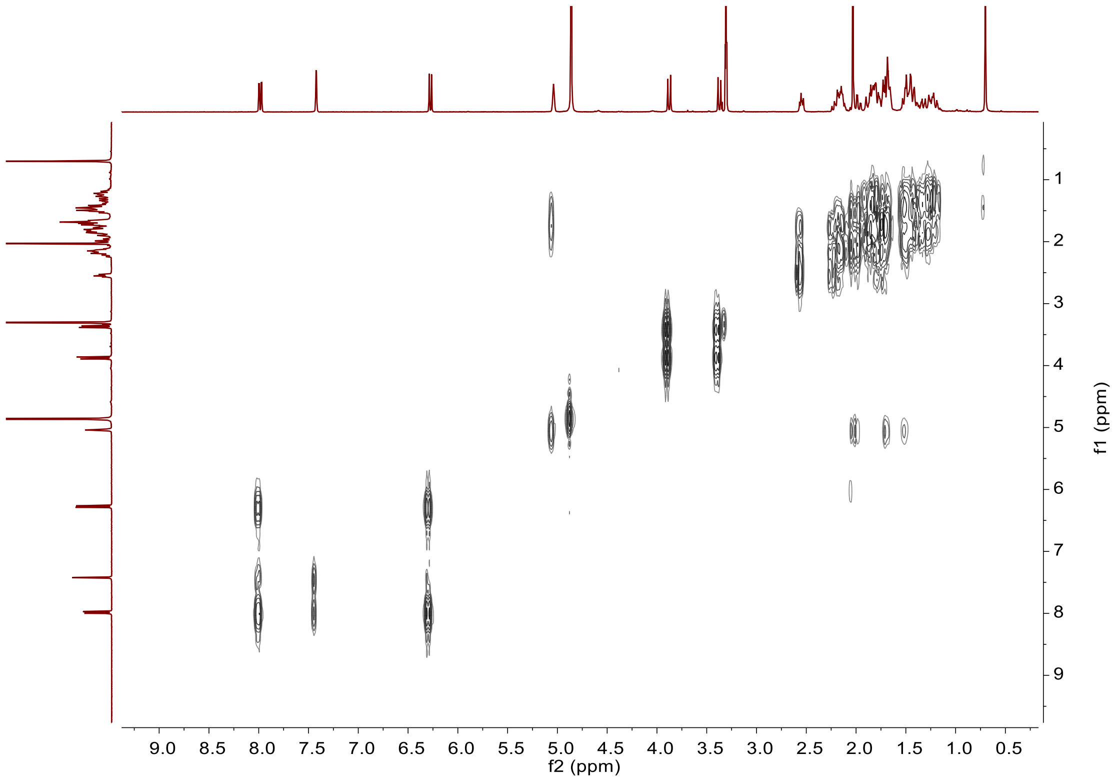

Figure S1.6. HSQC spectrum of **1** (in CD_3OD)

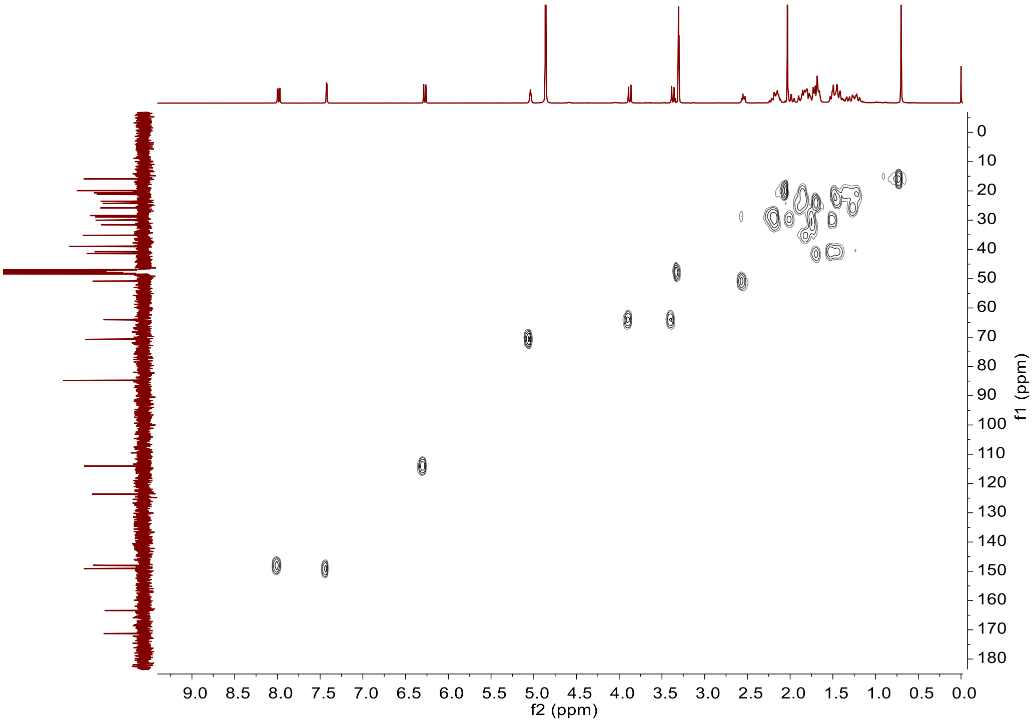

Figure S1.7. HMBC spectrum of **1** (in CD_3OD)

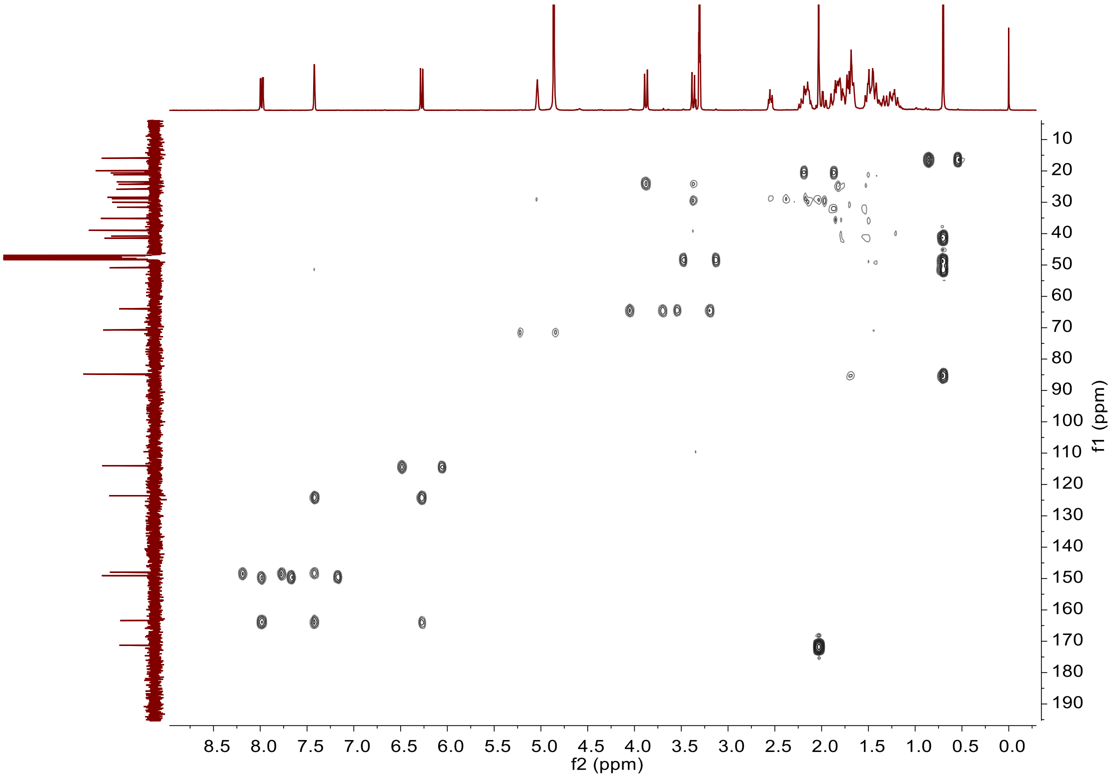

Figure S1.8. NOESY spectrum of **1** (in CD_3OD)

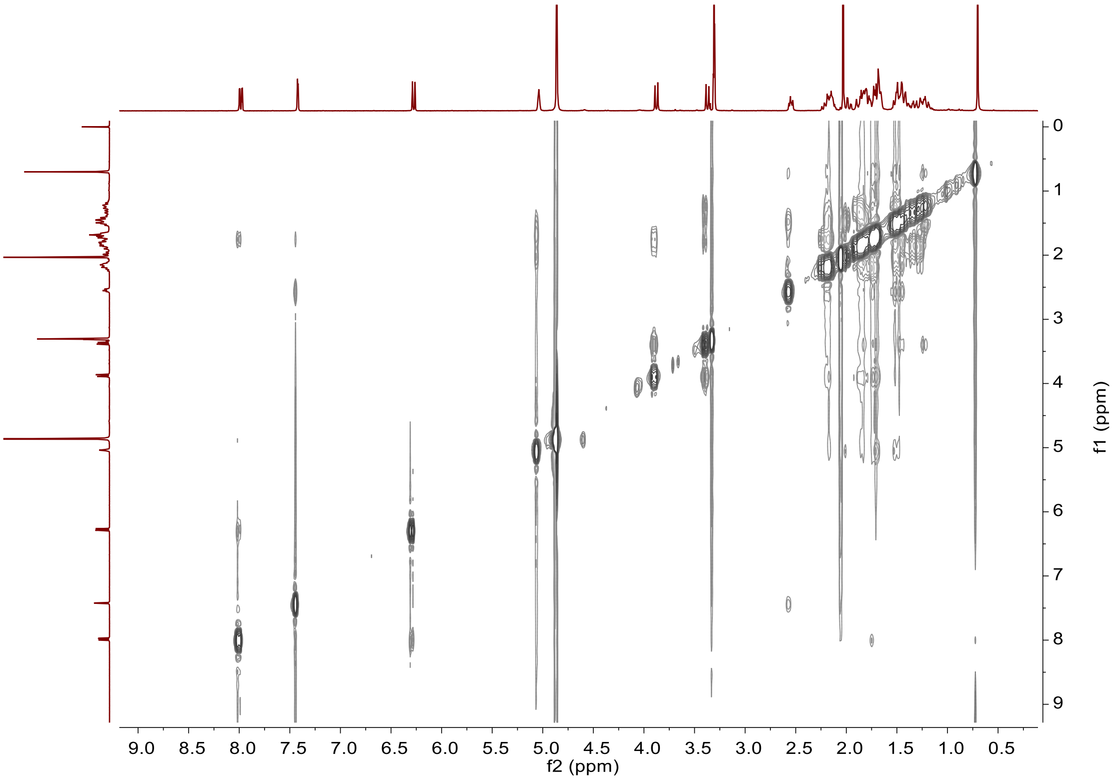

Figure S1.9. HR-ESI-MS spectrum of **2**

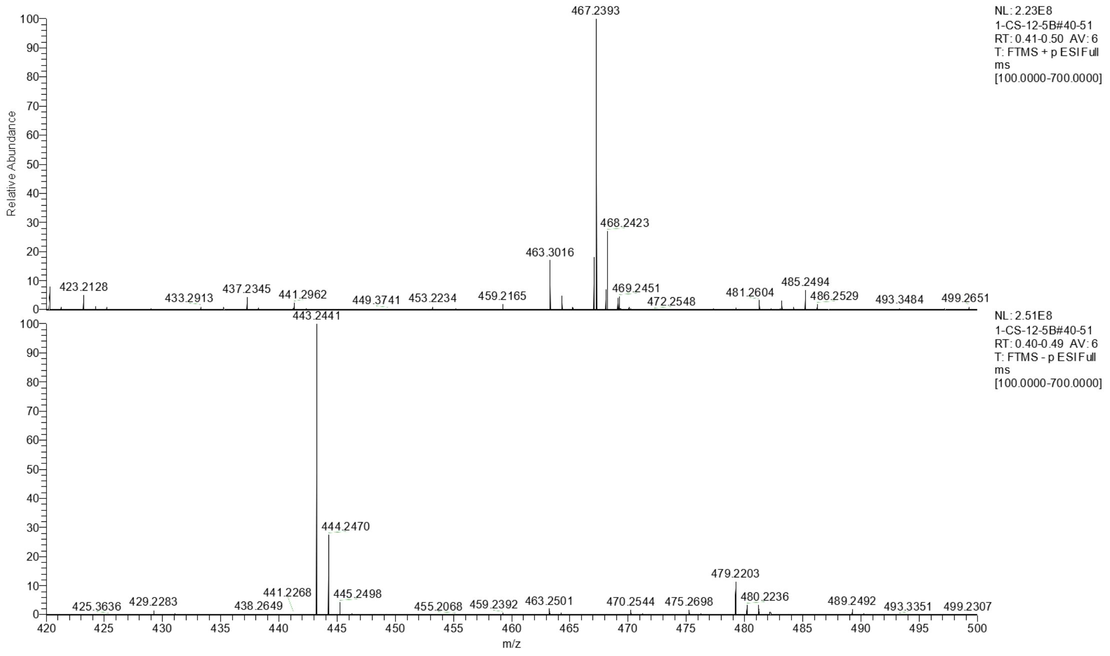

Figure S1.10. ^{1}H NMR spectrum of **2** (in CD_3OD, 400 MHz)

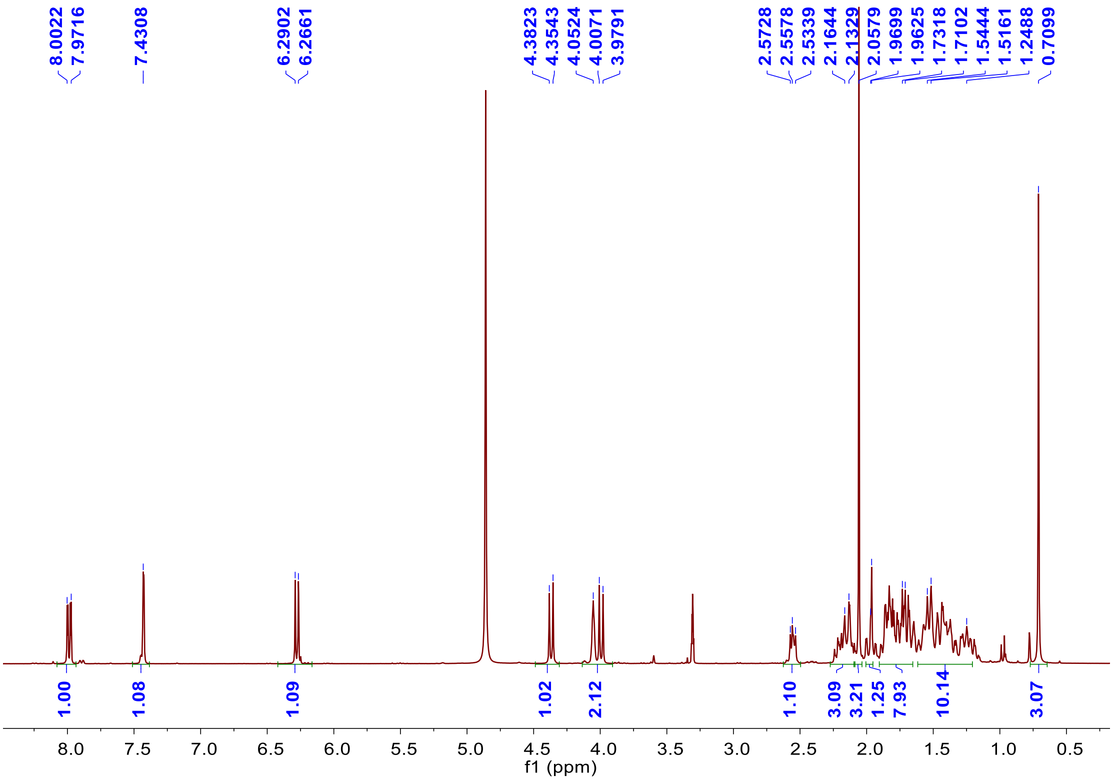

Figure S1.11. ^{13}C NMR spectrum of **2** (in CD_3OD, 100 MHz)

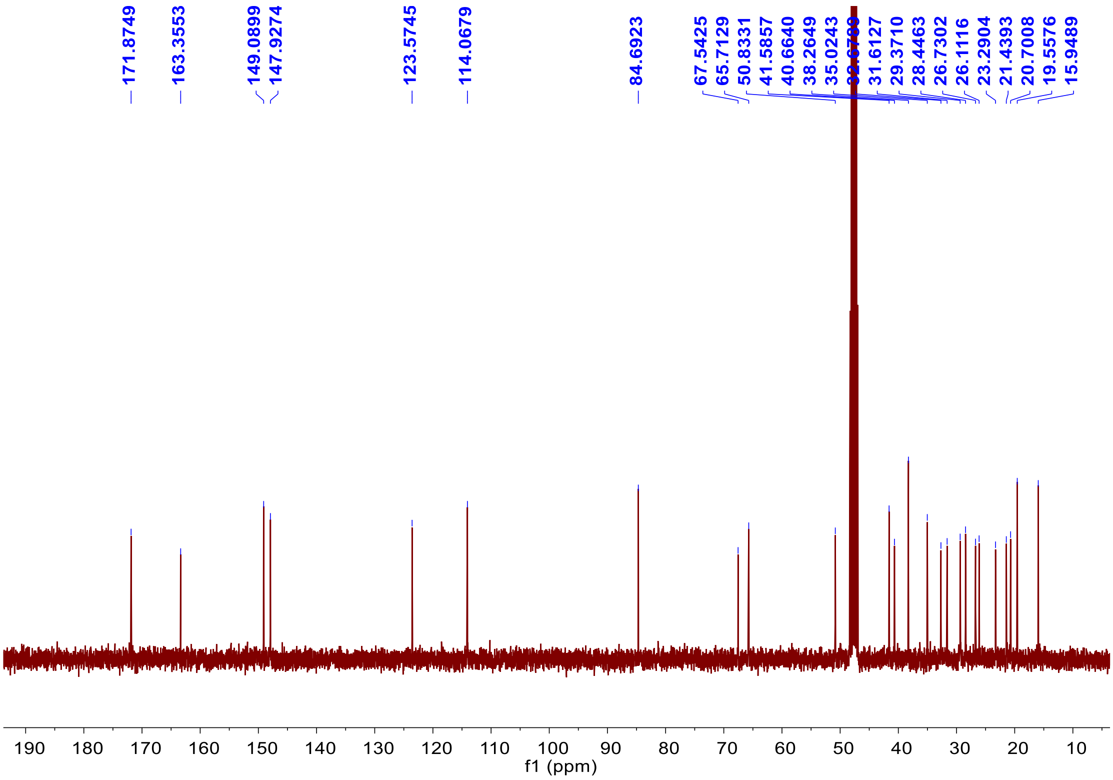

Figure S1.12. DEPT-135 spectrum of **2** (in CD_3OD, 100 MHz)

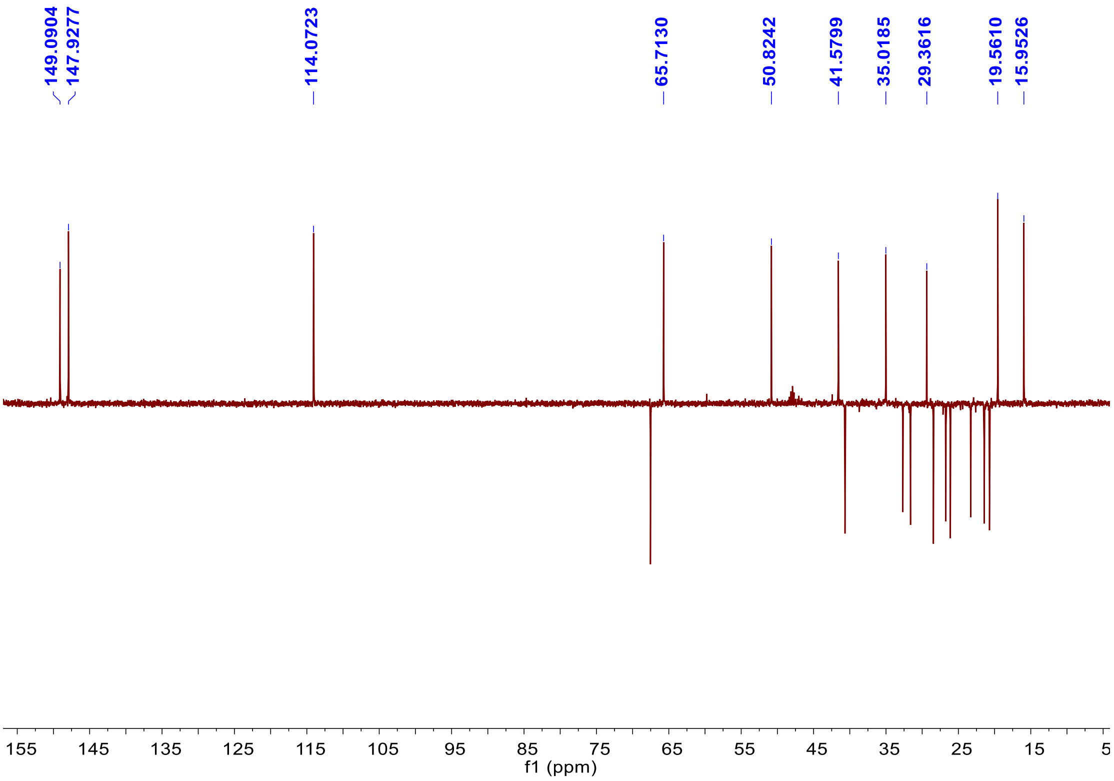

Figure S1.13. ^1H-^1H COSY spectrum of **2** (in CD_3OD)

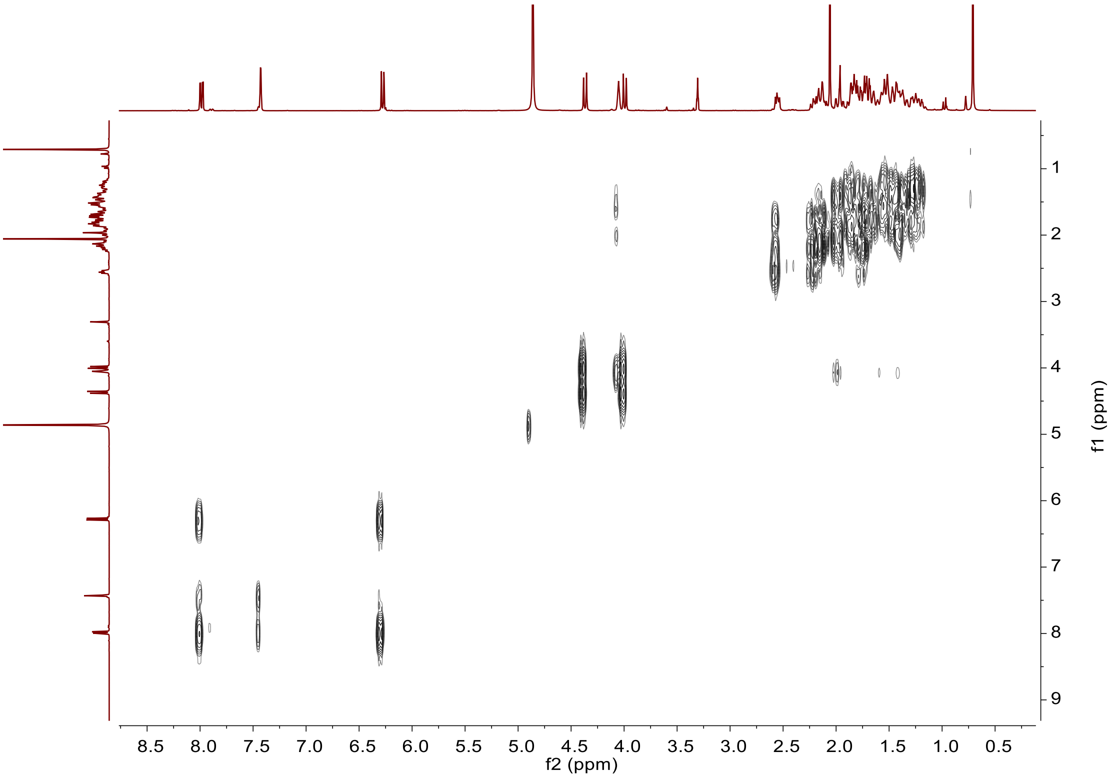

Figure S1.14. HSQC spectrum of **2** (in CD_3OD)

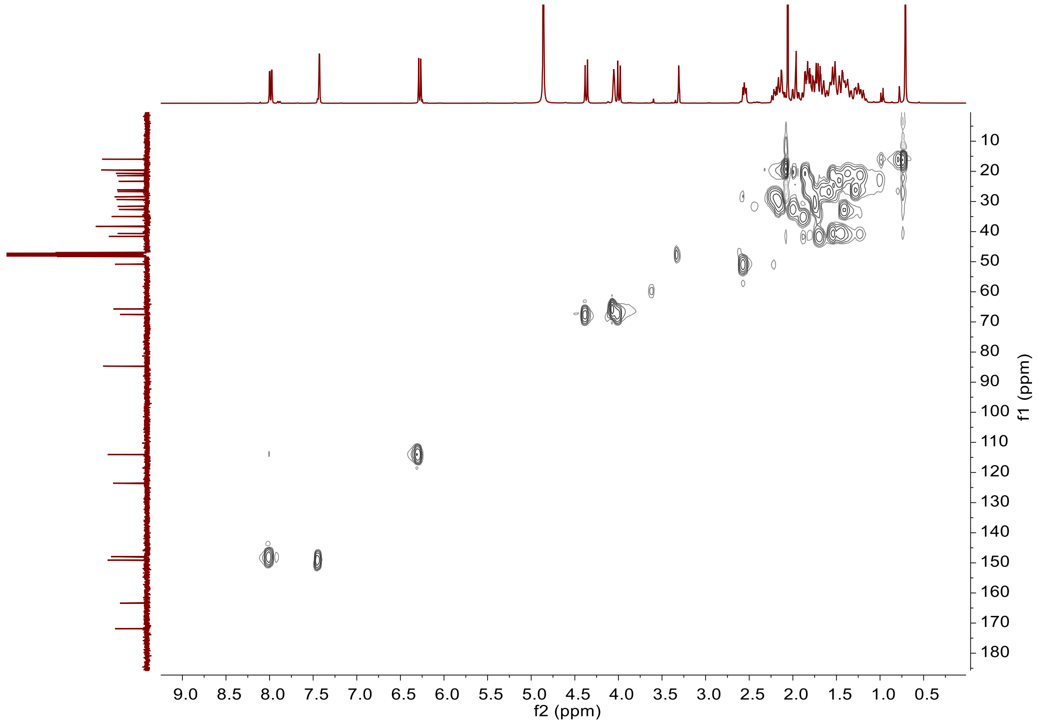

Figure S1.15. HMBC spectrum of **2** (in CD_3OD)

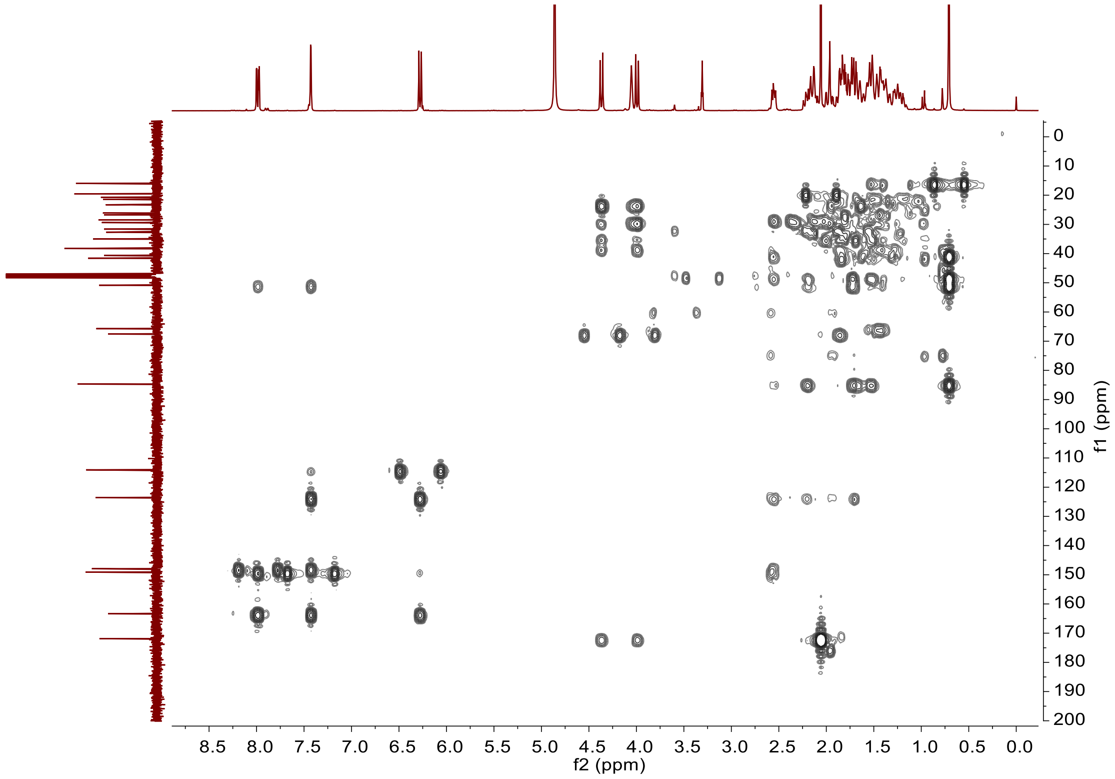

Figure S1.16. NOESY spectrum of **2** (in CD_3OD)

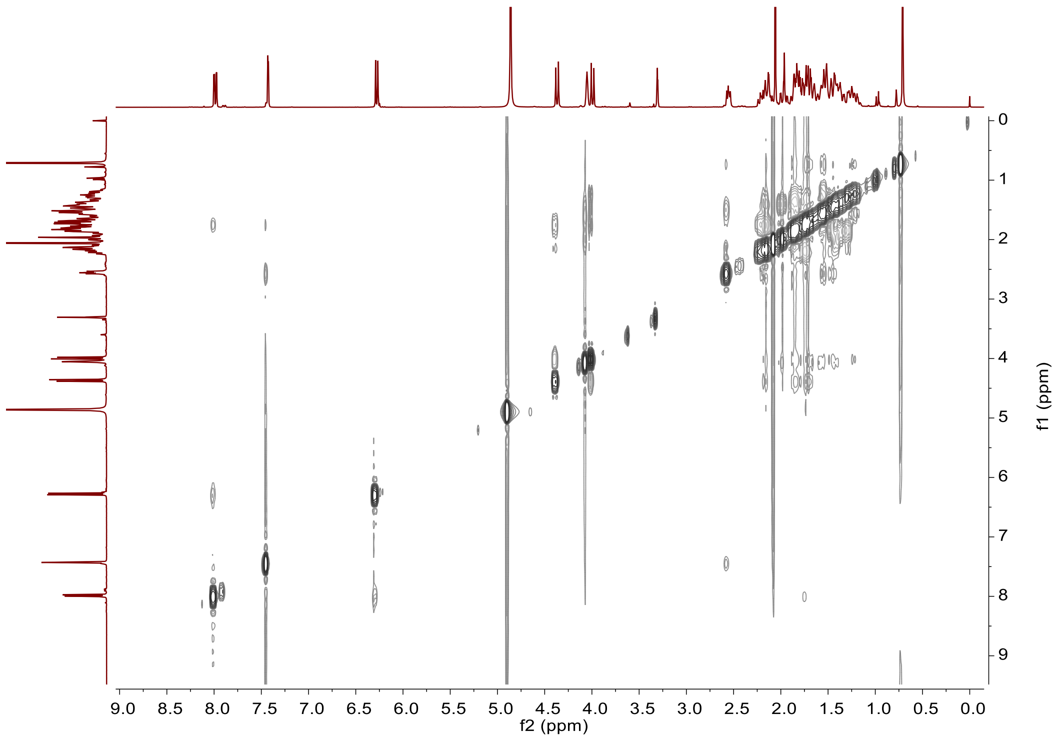

Figure S2. Spectra of four lactam (**14-17**) of bufadienolides by the conversion of lactone of bufadienolides

Figure S2.1. ^{1}H NMR spectrum of **14** (in CD_3OD, 600 MHz)

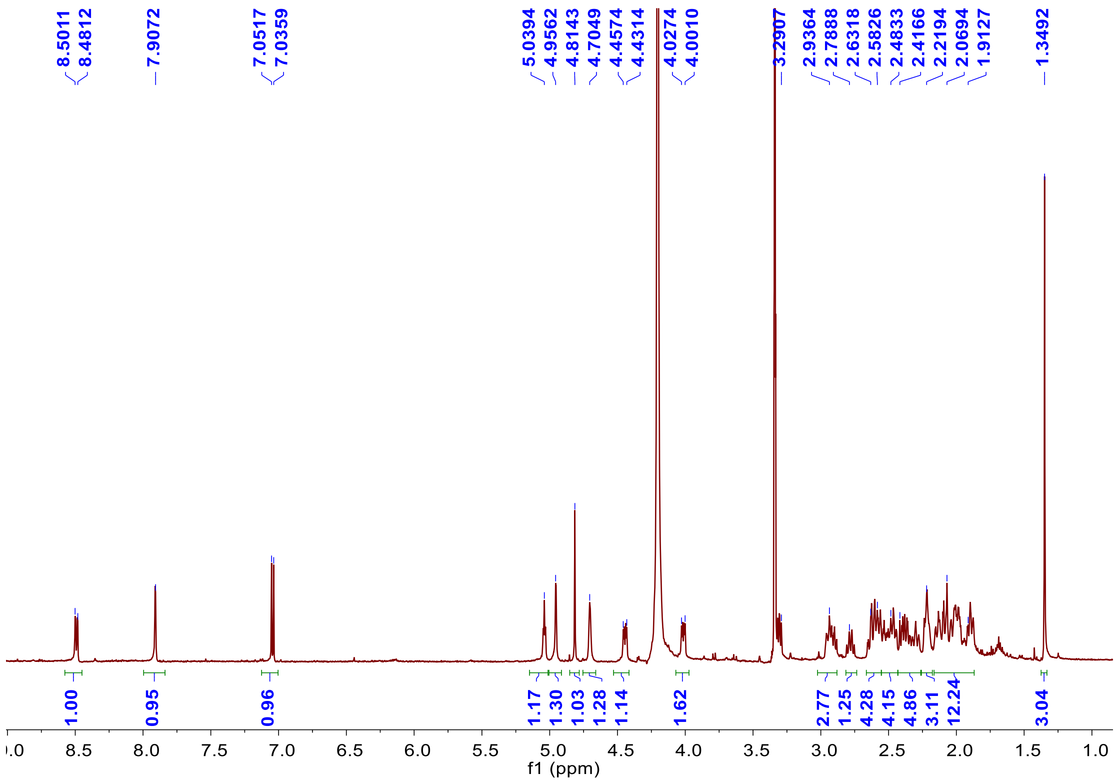

Figure S2.2. ^{13}C NMR spectrum of **14** (in CD_3OD, 150 MHz)

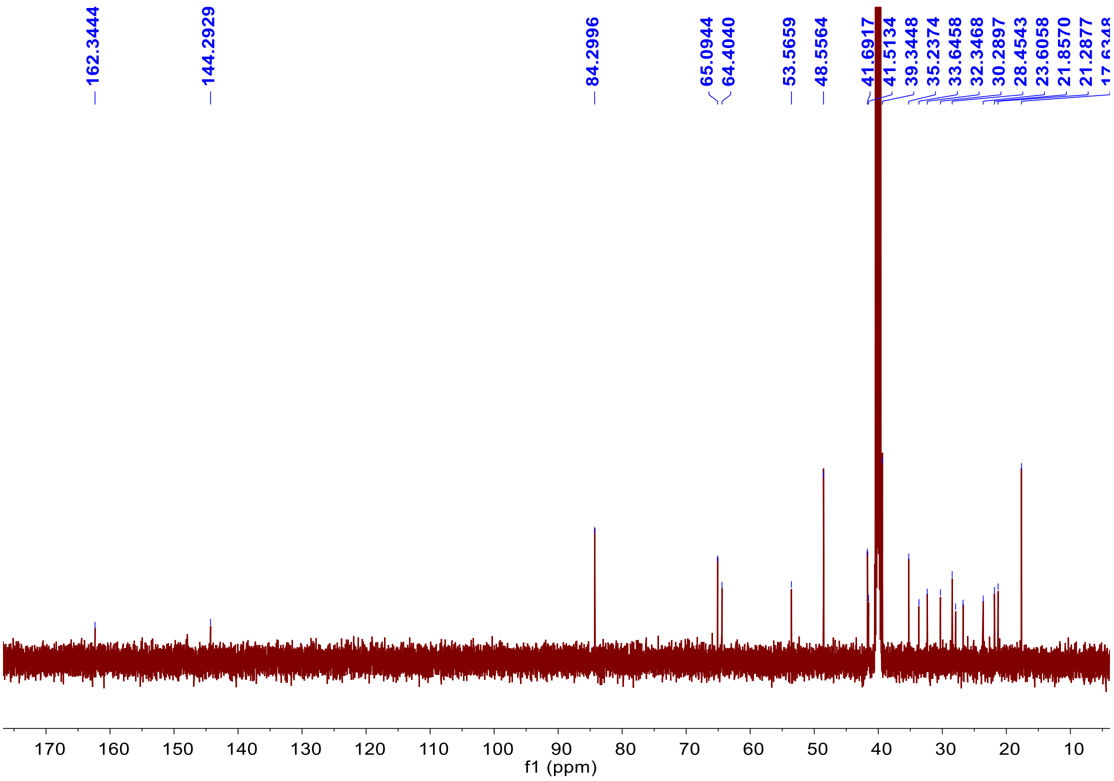

Figure S2.3. ^{1}H NMR spectrum of **15** (in CD_3OD, 600 MHz)

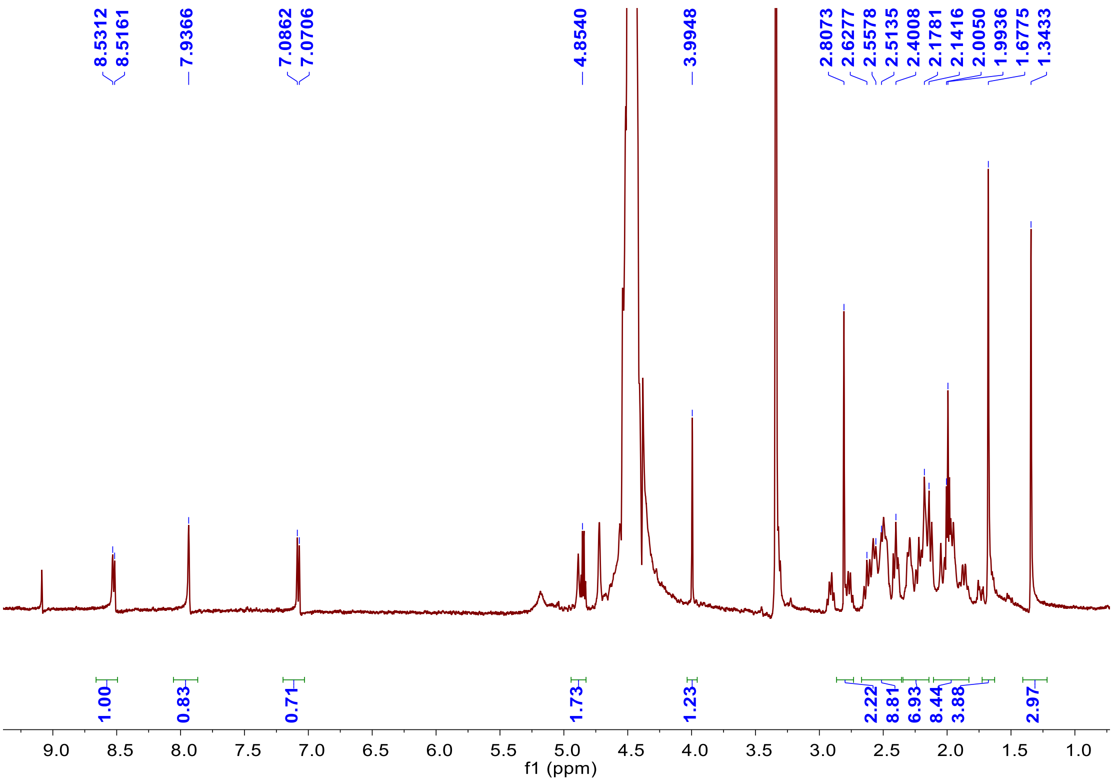

Figure S2.4. ^{13}C NMR spectrum of **15** (in CD_3OD, 150 MHz)

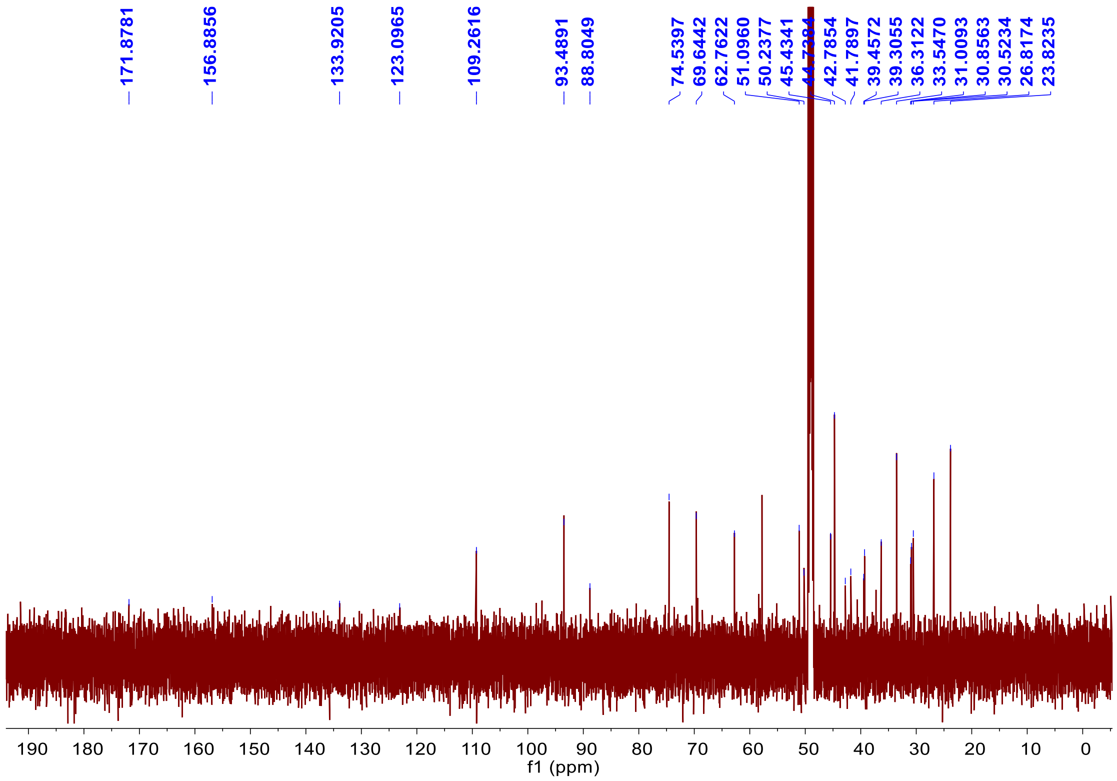

Figure S2.5. ^{1}H NMR spectrum of **16** (in CD_3OD, 600 MHz)

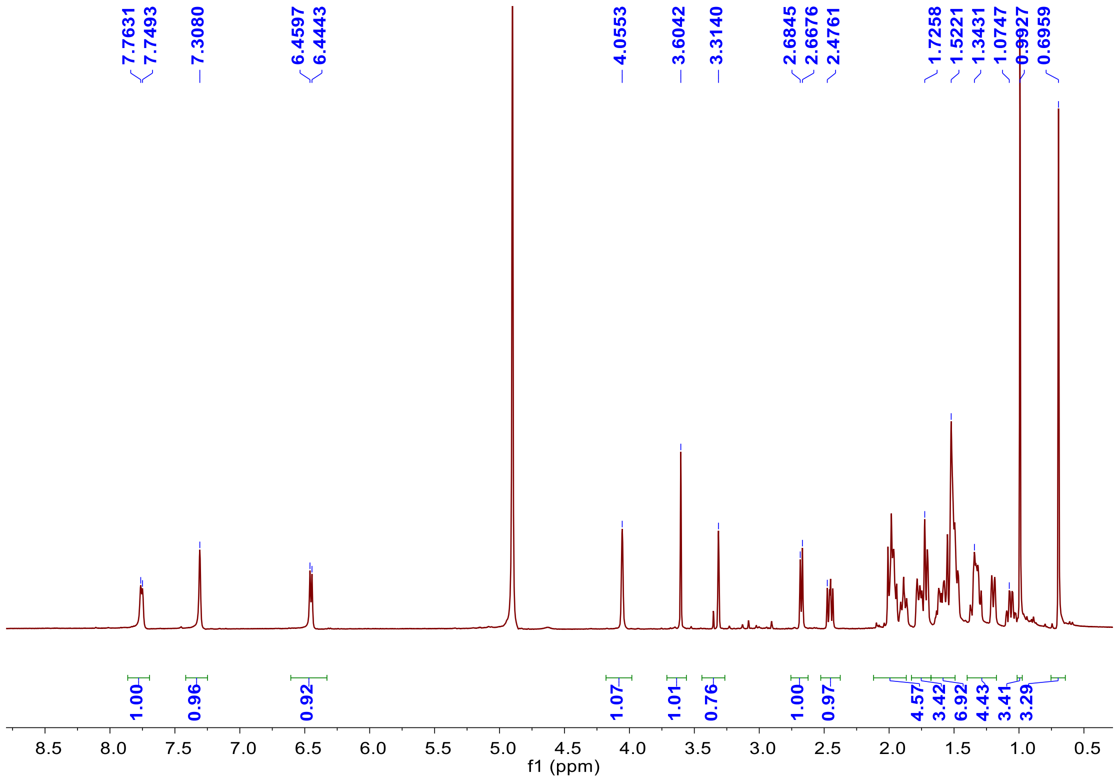

Figure S2.6. ^{13}C NMR spectrum of **16** (in CD_3OD, 150 MHz)

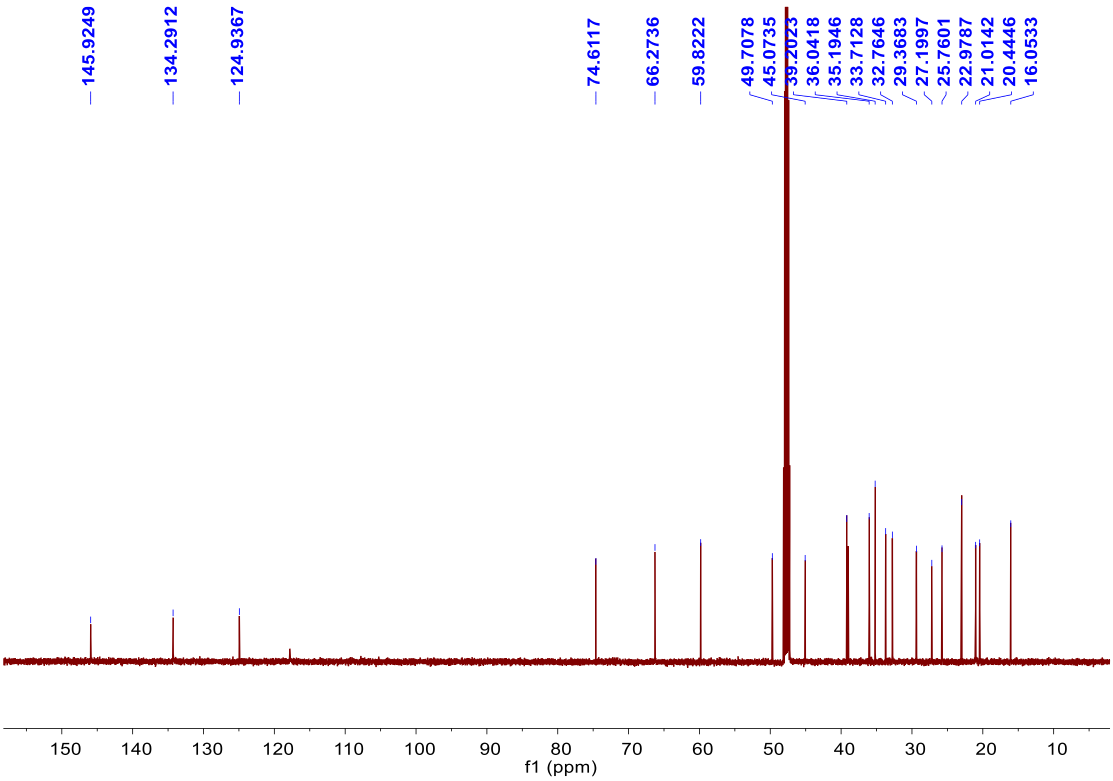

Figure S2.7. ^{1}H NMR spectrum of **17** (in CD_3OD, 600 MHz)

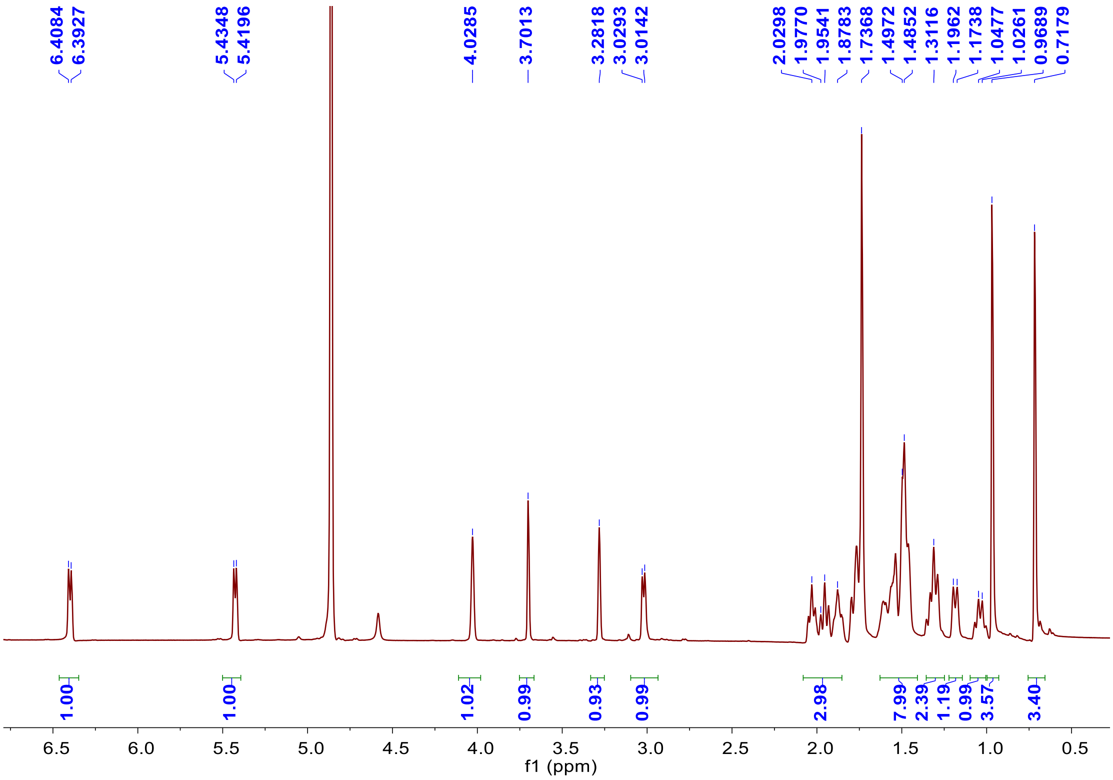

Figure S2.8. ^{13}C NMR spectrum of **17** (in CD_3OD, 150 MHz)

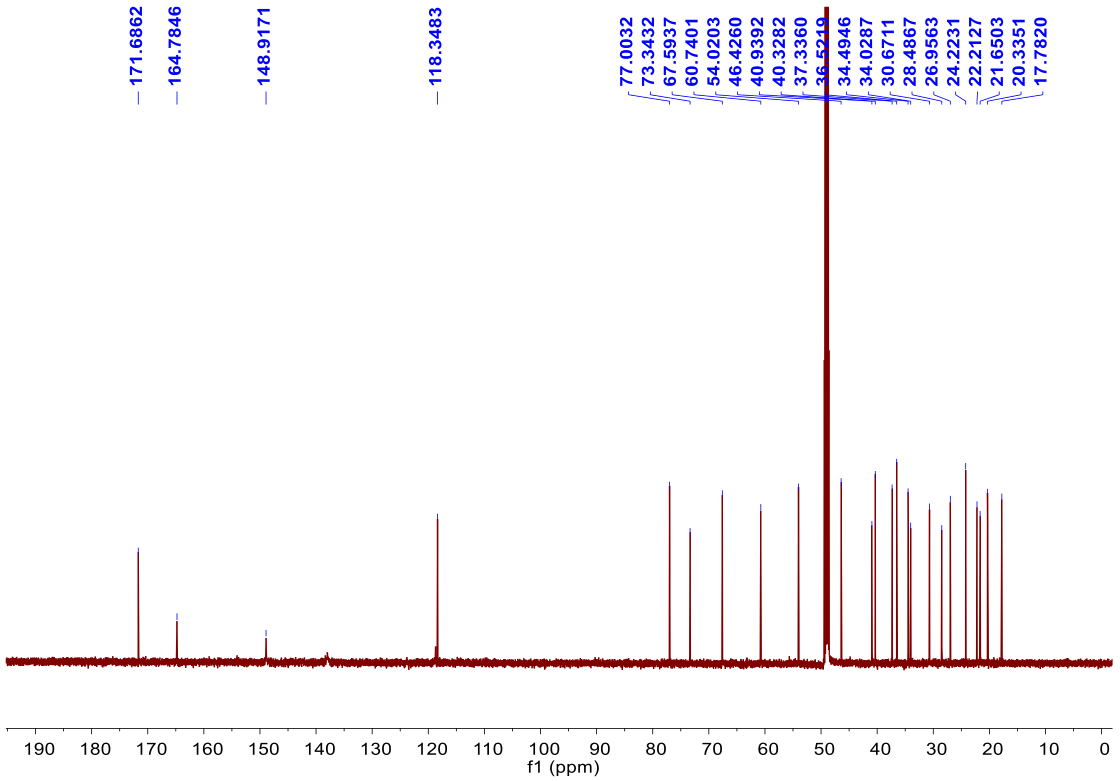

Table S1. ^{1}H (600 MHz) and ^{13}C (150 MHz) NMR spectroscopic data of **14** in CD_3OD

Position	19-Hydroxybufalin-lactam (**14**)	
	δ_C	δ_H (J in Hz)
1	26.2, CH_2	1.70, (overlapped); 1.03, (overlapped)
2	27.4, CH_2	1.40, (overlapped)
3	64.6, CH	3.86, (br s)
4	33.1, CH_2	1.76, (overlapped); 1.17, (overlapped)
5	27.9, CH	2.10, (overlapped)
6	23.1, CH_2	1.78, (overlapped); 1.13, (overlapped)
7	20.8, CH_2	1.73, (overlapped); 1.05, (overlapped)
8	41.2, CH	1.52, (overlapped)
9	34.7, CH	1.62, (overlapped)
10	38.8, C	-
11	21.4, CH_2	1.30, (overlapped); 1.05, (overlapped)
12	41.0, CH_2	1.38, (overlapped); 1.27, (overlapped)
13	48.1, C	-
14	83.8, C	-
15	31.8, CH_2	1.94, (overlapped); 1.56, (overlapped)
16	29.8, CH_2	2.07, (overlapped); 1.65, (overlapped)
17	53.1, CH	2.48, (overlapped)
18	17.1, CH_3	0.51, s
19	63.9, CH_2	3.60, dd (10.8, 4.8); 3.17, dd (10.8, 4.8)
20	122.3, C	-
21	133.4, CH	7.07, d (2.6)
22	143.8, CH	7.65, dd (9.5, 2.6)
23	118.6, CH	6.20, d (9.5)
24	161.9, C	-

Table S2. ^{1}H (600 MHz) and ^{13}C (150 MHz) NMR spectroscopic data of **15** in CD_3OD

Position	Bufalin-lactam (**15**)	
	δ_C	δ_H (J in Hz)
1	29.8, CH_2	1.36, (overlapped)
2	27.8, CH_2	1.47, (overlapped); 1.34, (overlapped)
3	65.1, CH	3.88, (br. s)
4	33.3, CH_2	1.81, (overlapped); 1.17, (overlapped)
5	36.0, CH	1.08, (overlapped)
6	26.8, CH_2	1.77, (overlapped); 1.13, (overlapped)
7	21.4, CH_2	1.30, (overlapped); 1.06, (overlapped)
8	41.6, CH	1.47, (overlapped)
9	35.3, CH	1.58, (overlapped)
10	35.3, C	-
11	21.0, CH_2	1.74, (overlapped); 1.12, (overlapped)
12	40.8, CH_2	1.38, (overlapped)
13	48.3, C	-
14	84.0, C	-
15	32.3, CH_2	1.93, (overlapped); 1.56, (overlapped)
16	30.0, CH_2	2.07, (overlapped); 1.65, (overlapped)
17	53.3, CH	2.48, (overlapped)
18	17.3, CH_3	0.50, s
19	24.1, CH_3	0.84, s
20	123.1, C	-
21	133.6, CH	7.10, s
22	144.4, CH	7.68, d (9.5)
23	118.7, CH	6.24, d (9.5)
24	162.4, C	-

Table S3. ^{1}H (600 MHz) and ^{13}C (150 MHz) NMR spectroscopic data of **16** in CD_3OD

Position	Resibufogenin-lactam (**16**)	
	δ_C	δ_H (J in Hz)
1	30.7, CH_2	1.50, (overlapped)
2	28.5, CH_2	1.55, (overlapped)
3	67.6, CH	4.02, (br. s)
4	34.1, CH_2	2.42, (overlapped); 1.30, (overlapped)
5	37.4, CH	1.73, (overlapped)
6	27.1, CH_2	1.86, (overlapped); 1.16, (overlapped)
7	21.8, CH_2	1.45, (overlapped); 1.04, (overlapped)
8	40.3, CH	1.73, (overlapped)
9	35.0, CH	1.95, (overlapped)
10	36.5, C	-
11	22.3, CH_2	1.53, (overlapped); 1.30, (overlapped)
12	40.5, CH_2	1.52, (overlapped)
13	46.4, C	-
14	75.9, C	-
15	61.1, CH_2	3.57, (overlapped)
16	34.1, CH_2	1.96, (overlapped)
17	51.0, CH	2.65, d (10.2)
18	17.4, CH_3	0.67, s
19	24.3, CH_3	0.96, s
20	126.3, C	-
21	135.6, CH	7.28, s
22	147.2, CH	7.73, d (9.4)
23	119.1, CH	6.42, d (9.4)
24	162.4, C	-

Table S4. ^{1}H (600 MHz) and ^{13}C (150 MHz) NMR spectroscopic data of **17** in CD_3OD

Position	Cinobufagin-lactam **(17)**	
	δ_C	δ_H (J in Hz)
1	30.7, CH_2	1.50, (overlapped)
2	28.5, CH_2	1.63, (overlapped)
3	67.6, CH	4.04, (br. s)
4	34.0, CH_2	1.95, (overlapped); 1.33, (overlapped)
5	37.3, CH	1.76, (overlapped)
6	27.0, CH_2	1.80, (overlapped); 1.20, (overlapped)
7	21.7, CH_2	1.47, (overlapped); 1.04, (overlapped)
8	40.3, CH	2.05, (overlapped)
9	34.5, CH	1.73, (overlapped)
10	36.5, C	-
11	22.2, CH_2	1.54, (overlapped); 1.33, (overlapped)
12	40.9, CH_2	1.82, (overlapped); 1.54, (overlapped)
13	46.4, C	-
14	73.3, C	-
15	60.7, CH_2	3.71, d (3.4)
16	77.0, CH_2	5.43, dd (9.7, 3.3)
17	54.0, CH	3.03, (overlapped)
18	17.8, CH_3	0.72, s
19	24.2, CH_3	0.97, s
20	118.7, C	-
21	138.0, CH	7.23, (br. s)
22	148.9, CH	7.85, (br. s)
23	118.4, CH	6.40, dd (9.7, 3.5)
24	164.8, C	-
25	171.7, C	-
26	20.3, CH_3	1.74, s
